# Aqueous Integrated Process for the Recovery of Oil Bodies or Fatty Acid Emulsions from Sunflower Seeds

**DOI:** 10.3390/biom12020149

**Published:** 2022-01-18

**Authors:** Audrey Cassen, Jean-François Fabre, Eric Lacroux, Muriel Cerny, Guadalupe Vaca-Medina, Zéphirin Mouloungui, Othmane Merah, Romain Valentin

**Affiliations:** Laboratoire de Chimie Agro-Industrielle (LCA), Université de Toulouse, INRAE, INPT, 31030 Toulouse, France; audrey.cassen@ensiacet.fr (A.C.); eric.lacroux@ensiacet.fr (E.L.); muriel.cerny@ensiacet.fr (M.C.); Guadalupe.VacaMedina@ensiacet.fr (G.V.-M.); zephirin.mouloungui@ensiacet.fr (Z.M.); othmane.merah@ensiacet.fr (O.M.); romain.valentin@ensiacet.fr (R.V.)

**Keywords:** fatty acids, extraction, Cryo-SEM, oleosomes, phospholipids

## Abstract

An aqueous integrated process was developed to obtain several valuable products from sunflower seeds. With a high-shear rate crusher, high-pressure homogenization and centrifugation, it is possible to process 600× *g* of seeds in 1400× *g* of water to obtain a concentrated cream phase with a dry matter (dm) content of 46%, consisting of 74 (*w*/*w* dm) lipids in the form of an oil-body dispersion (droplet size d(0.5): 2.0 µm) rich in proteins (13% *w*/*w* dm, with membranous and extraneous proteins). The inclusion of an enzymatic step mediated by a lipase made possible the total hydrolysis of trigylcerides into fatty acids. The resulting cream had a slightly higher lipid concentration, a ratio lipid/water closer to 1, with a dry matter content of 57% consisting of 69% (*w*/*w*) lipids, a more complex structure, as observed on Cryo-SEM, with a droplet size slightly greater (d(0.5): 2.5 µm) than that of native oil bodies and a conserved protein concentration (12% *w*/*w* dm) but an almost vanished phospholipid content (17.1 ± 4.4 mg/g lipids compared to 144.6 ± 6 mg/g lipids in the oil-body dispersion and 1811.2 ± 122.2 mg/g lipids in the seed). The aqueous phases and pellets were also characterized, and their mineral, lipid and protein contents provide new possibilities for valorization in food or technical applications.

## 1. Introduction

Seed oil bodies (OBs), also known as oleosomes, are plant intracellular organelles. They have a specifically organized molecular structure consisting of a triglyceride (TAG) core surrounded by a phospholipid (PL) monolayer and proteins [1,2,3], and act as natural surfactant molecules, with the hydrophobic fatty acid moiety of the PL facing the core of the oil droplet and the hydrophilic polar head group oriented towards the cellular environment. The structure of the membranous proteins and their interactions with the PL monolayer, the TAG matrix and the external cellular medium have been studied in detail, and it was demonstrated that the synergetic action of phospholipids and proteins is responsible for the high stability of oil bodies [4,5]. The OBs are major food reserves used by the plant to drive germination and post-germination growth. They are, therefore, considered to be a potentially useful source of lipids and proteins. The most widely used solvent for lipid extraction from seeds is hexane, even in the food industry. This solvent is highly toxic, flammable, and represents a real danger to health and the environment. For this reason, the phasing out of hexane use and its replacement by aqueous extraction methods for lipid extraction from oilseeds is a subject of major interest internationally, with intensive development of this technological approach. Most aqueous oil extraction (AOE) methods are based on the use of enzymes. Cellulases are often used to degrade cell walls, and proteases favor lipid release by removing the proteins at the surface of the oil body. These enzymes can also be used in conjunction with a pectinase, an enzyme that degrades polysaccharides. Even if they are generally exogenous to the seed, in some processing conditions, the expression of endogenous enzymes is possible and may be beneficial for oil release [4].

Such enzyme-assisted AOE methods, sometimes combined with protein extraction, have been studied and applied to different kinds of oilseeds: rapeseed [5,6], canola [7], sunflower [8,9,10,11], sesame [12], maize [13], groundnut [4], almond [14] and safflower [15], but the most studied matrix is undoubtedly soybean [16,17,18,19,20]. Many studies have reported the development of ultrasound-assisted AOE [21,22,23,24,25,26,27,28], but other methods are also used, including microwave- [29], pulsed electric field-[9], pressure shockwave- [30] and surfactant-assisted [31,32] extraction. Unlike conventional extraction with an organic solvent, aqueous extraction from oilseeds often gives rise to emulsions, and the recovery of lipids and proteins with good yields involves a de-emulsification step leading to disorganization of the inherent structure of the oil body [24,33,34]; however, the use of OBs as a formulated emulsion may be of considerable utility, as emulsions of this type have a remarkable physical stability and contain many valuable natural compounds, such as phospholipids and tocopherols. Several studies have recently focused on isolated OBs, highlighting their physical stability [35,36], organization and functional properties [13,35,36,37,38] and their interactions with other substances [39]. Moreover, a specific study of the lipidome and proteome of OBs isolated from sunflower highlighted the molecular profile of such organized systems [40]. Oil bodies are used principally in food applications; their natural stability and advantageous protein and lipid compositions directly suggest uses in many products of four principal types [41]. They can be used as imitation milk products, such as soymilk [42] and mayonnaise [43], as natural emulsions for encapsulating flavors, for example [44], as natural emulsifiers, given their behavior at interfaces [45], and as oil-body-based edible films [46].

We describe here a novel process for releasing oil bodies. The main process results in the release of oil bodies by dispersion in water, resulting in an oil-in-water emulsion in which the oil droplets are the native oil bodies of the seed. The originality of this process lies first in the release of the OBs by upscalable purely mechanical means into an aqueous medium, without the assistance of a surfactant or enzyme and secondly in its versatility as the use of a high-pressure homogenization allows both particle size reduction and the improved action of an exogenous lipase to form a complex fatty acid (FA) emulsion. We obtained a global view of these processes by characterizing and comparing the emulsions and all the fractions collected at the end of the main and modified processes.

## 2. Results and Discussion

### 2.1. Integrated Process for the Release of Sunflower Seed Olesomes

**Figure 1:**
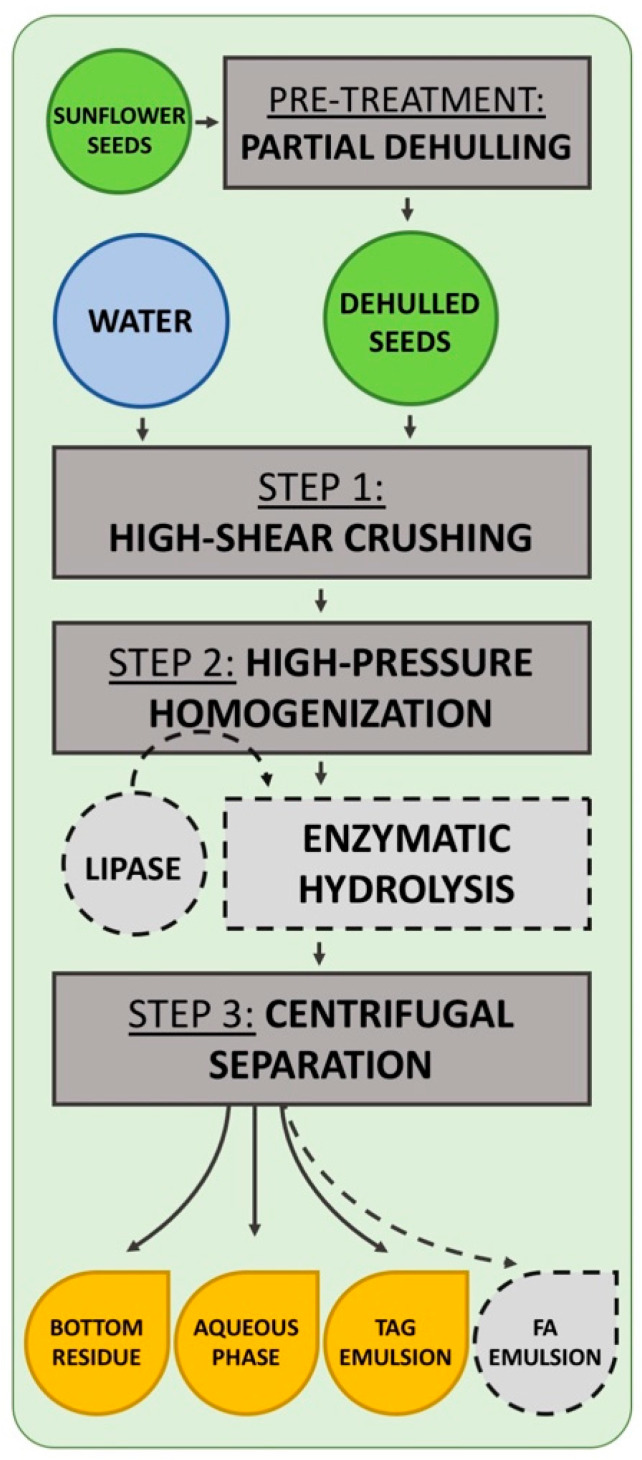
Schematic diagram of the integrated process of lipid extraction from sunflower seeds.

The lignocellulosic shell accounts for a large proportion of the sunflower seed. Integrated aqueous transformation processes therefore begin with a partial dehulling step (Figure 1, pretreatment). This mechanical shelling makes it possible to prevent the flotation phenomena generated by the presence of a very high proportion of shells, which can disrupt shearing with a rotor/stator.

Subsequent steps in the process are designed to promote the release of the lipid bodies present in the seeds. Thus, in Step 1 (Figure 1), the shelled seeds are crushed in water with a high-shear rotor/stator-type mill. This initiates the release of the lipid bodies into the water in the form of an emulsion-type suspension. Some of the lipid bodies are released from the seed matrix, remaining in their native state. The presence of the water makes it possible for them to remain in suspension, without their structure being destroyed, which is not possible with dry grinding. At the macroscopic scale, this step (Step 1, Figure 1) allows us to pass from the initial size of the seeds (8 to 11 mm before shelling, 4 to 10 mm after shelling) to a mean particle size in suspension of 0.5 to 1 mm. At this stage, the seed constituents are in a hydrated state and display very strong aggregation, particularly for the lipid bodies.

Step 2 therefore involves gradually increasing the shearing phenomena to promote the release of lipid bodies and lignocellulosic constituents by reducing the interactions between them. High-pressure homogenization is the most appropriate technological treatment for this purpose. The main objective of this step is to reduce the size of the particles obtained in Step 1 by promoting hydration, to optimize the release of lipid bodies from the lignocellulosic matrix, thereby quantitatively optimizing the yield of released lipids. This step involves 3 stages of pressure increase, as described in the experimental procedures section, to produce a medium consisting of lignocellulosic particles with a diameter of between 50 and 100 μm and lipid droplets with a diameter of between 1 and 2.5 μm.

Catalytic elements, such as lipases, can be introduced during Step 2 for the hydrolysis of triglycerides into lipophilic free fatty acids and hydrophilic glycerol. High-pressure homogenization results in the dispersion of the lipase throughout the medium, while promoting the oil/water contact surface, in turn promoting the interfacial catalytic activity of the lipase. After homogenization, the suspension can be incubated with mechanical agitation, for however long is required to complete the hydrolysis of the triglycerides present in the sunflower oil.

This enzymatic transformation of the triglycerides present in the lipid bodies transforms the structure of the oil bodies, resulting in a transition from an oil-in-water type emulsion to a complex oil-in-water-in-oil and/or water-in-oil-in-water emulsion.

The final step of the process (Step 3) involves a centrifugal separation of the phases based on differences in density. The upper phase, consisting of the lipid bodies, which can be considered an oil-in-water emulsion phase, can be separated from the aqueous middle phase, which is rich in soluble trace elements. The lower phase, consisting of the residual pellet, is composed of the densest lignocellulosic elements.

The upper creamy layer is an oil-in-water emulsion corresponding to 8% (*w*/*w*) of the total matter recovered at the end of the process. The internal phase of the oil droplets is mainly composed of triglycerides and this phase can be named “triglyceride emulsion” in an abbreviated way. An aqueous phase accounting for 54% (*w*/*w*) of the total matter recovered, and a bottom residue rich in lignocellulosic materials (38% *w*/*w*) is also recovered. The integrated process is performed in a non-buffered medium. The seeds are, therefore, crushed at the native pH of 6.3. This pH is close to the isoelectric point of the proteins, which is estimated at between 5 and 6 for oleosin proteins [35] and at about 5.5 for water-soluble proteins [47]. The droplets are, therefore, easily flocculated. It is therefore possible to use reasonably low centrifugation speeds to concentrate the lipids in the cream phase.

### 2.2. Composition of Each Fraction

All studies on OB recovery to date have focused on characterizing the components of the emulsion. A global vision of the whole process for obtaining these emulsions was lacking, and the by-products were not considered. In this study, we determined of the composition of each fraction (Table 1).

The extraneous matter was determined by difference: 100-(moisture + ash + lipid + protein). It consisted mostly of polysaccharides, fibers and other minor components, such as phospholipids, which are not characterized in detail here. The emulsion contained about 26% (*w*/*w*) of total seed lipids, a value close to the results obtained for soybean with a different aqueous extraction process [48]. Triglycerides generally account for about 95% of the total weight of sunflower oil bodies [3]; however, proteins account for more than 10% (*w*/*w*) of the total dry weight of the oil-body emulsion, indicating that the emulsion contains more than just the normal amount of oleosins. There is a concentration, by about 1%, of proteins in the aqueous phase, and the additional proteins present in the emulsion cannot be accounted for solely by the proteins brought by the aqueous phase of the emulsion. The elimination of these non-membranous proteins generally requires a harsh treatment of sunflower oil bodies with urea [49]. These co-extracted proteins are linked, to a certain extent, to the oil bodies, and they may form a second layer around the oil-body surface [13]. These proteins enhance the synergic behavior of phospholipids/oleosins in preventing coalescence and should remain in the cream to increase its physical stability [5].

### 2.3. Modification of the Integrated Process to Obtain a New Complex Fatty Acid Emulsion

The integrated process described above was modified by adding an enzymatic triglyceride hydrolysis step to obtain a new kind of emulsion. This hydrolysis was performed by a *Candida rugosa* lipase. The enzyme was blended with the medium before homogenization, to promote an intimate interfacial interaction between the lipase and oil bodies.

In optimal conditions, this enzyme can hydrolyze ester bonds and release glycerol and free fatty acids (FAs) into the medium. Hence, the internal lipids of the droplets are mainly free FAs and we thus obtain a “fatty acid emulsion”. To our knowledge, this is the first time a FA emulsion has been obtained directly by the in situ transformation of seeds. At the end of the process, the recovery of the three fractions was identical to that for the initial unmodified process, with a complex FA emulsion as a top creamy layer, together with an aqueous phase and a bottom residue. The complex FA emulsion accounted for 9% (*w*/*w*) of the total medium, whereas the aqueous phase and the bottom residue accounted for 54% (*w*/*w*) and 37% (*w*/*w*), respectively. Table 2 shows the composition of each fraction. The FA emulsion was more concentrated than the TAG emulsion, with fewer minerals and more lipids. The ratio of water/lipids (*w*/*w*) is lower than in the TAG emulsion (1.1 compared to 1.6). The total nitrogen and calculated protein contents were higher than for the process without the hydrolysis step, but this difference was not significant, and could be explained by the amount of enzyme used for hydrolysis. Moreover, there was clearly much more extraneous matter. We hypothesize that the extraneous matter included the glycerol released by hydrolysis, which was not determined here. This glycerol would also be present in the aqueous phase. A large amount of lipid was present in the bottom residue for both processes. In the enzymatic process, the fatty acid emulsion contained about 33% (*w*/*w*) of the lipids present in the seed, a slight but significantly higher proportion than was present in the triglyceride emulsion (26% (*w*/*w*)). The lipase may have facilitated the release of destabilized oil bodies by disrupting some lipoprotein linkages.

### 2.4. Observation of Sunflower Seeds and OBs by Cryo-SEM and Determination of the Size Distribution

**Figure 2:**
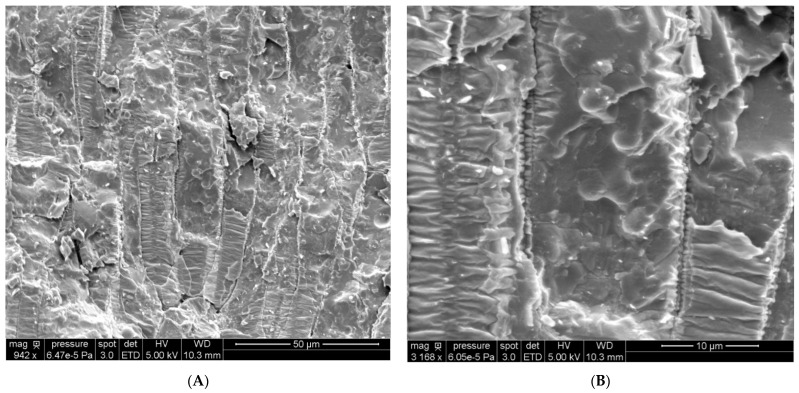
Cryo-SEM images of a sunflower seed prepared by the freeze-fracture technique. (**A**): cellular structure of the endosperm, (**B**): detail of a cell.

The mean size and size distribution of oil bodies in sunflower seeds have been extensively studied ([2,3]). Cryo-SEM experiments with freeze-fracture can be performed to observe the inner cellular structures (Figure 2).

**Figure 3:**
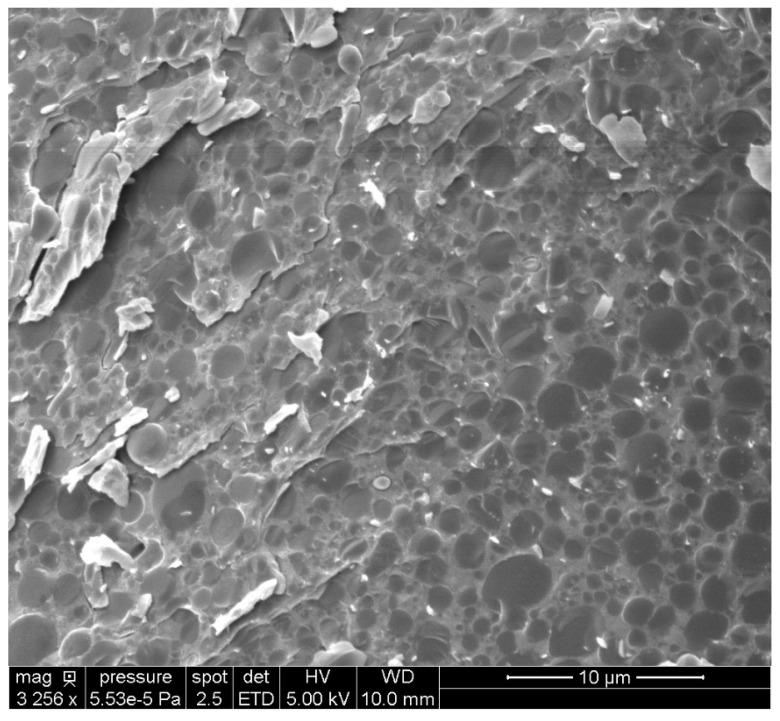
Cryo-SEM image of the triglyceride oil-in-water emulsion prepared by the freeze-fracture technique.

In partially opened cells, some intact spheres with a measured mean diameter close to 2.5 µm were observed. These spheres may correspond to the food reserves of the seed, the native OBs. The triglyceride oil-in-water emulsion was also observed by Cryo-SEM (Figure 3).

Oil droplets surrounded by the aqueous phase are clearly visible, and this observation is consistent with the findings of Nikiforidis et al. for maize germ and sunflower seed emulsions [50].

**Figure 4:**
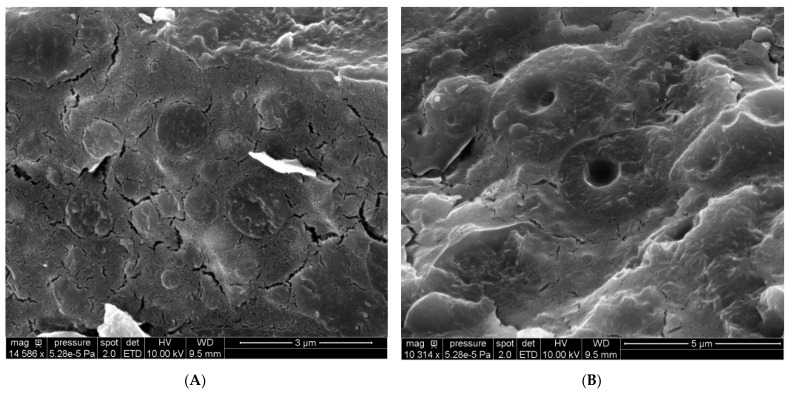
Cryo-SEM images of the complex fatty acid emulsion prepared by the freeze-fracture technique. (**A**): View of different droplets, (**B**): View of droplets embedded in other droplets.

Cryo-SEM images revealed that the FA emulsion had a complex structure (Figure 4).

Droplets with a diameter close to 2 µm were also observed. Unlike the triglyceride droplets observed by the same technique and in the same conditions, the inner part of the droplets appeared to be less smooth, with some relief observable. At higher magnification, it was possible to observe some spheres and holes in the droplets due to the freeze-fracture sampling technique. This observation demonstrates the complexity of this new kind of emulsion. 

**Figure 5:**
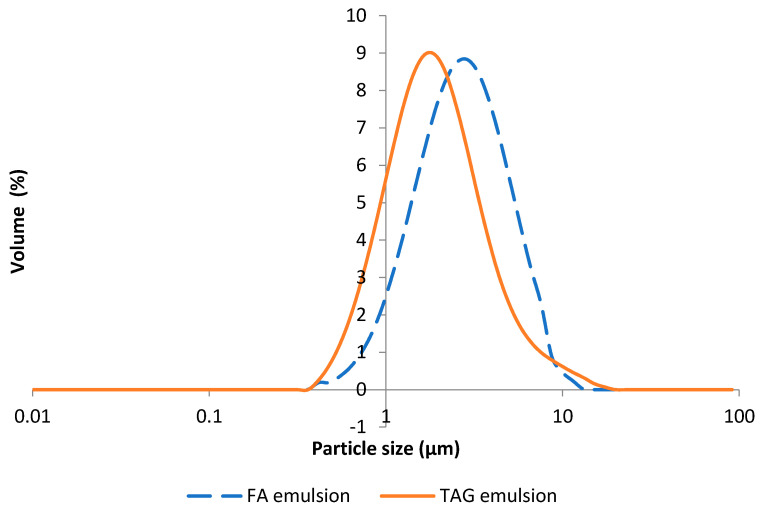
Size distribution of droplets from the fatty acid (FA) and triglyceride (TAG) emulsions.

Granulometric experiments revealed a broad size distribution of the OBs in the triglyceride oil-in-water emulsion (Figure 5).

The mean diameter (D[4,3]) was 2.6 µm ± 0.1 and the median diameter (d(0.5)) was 2.0 µm ± 0.1, consistent with the usual range for native OBs, as reported by Tzen et al. [2,3]. No significant coalescence phenomenon was observed in this emulsion, suggesting that the oil bodies were stable. Contrary to the findings of previous studies, our results show that it is possible to release sunflower seed OBs in their native form through the exclusive use of a mechanical process, in an aqueous medium, without the need for an enzyme or surfactant.

The mean size (D[4,3]) of the droplets in the FA emulsion was 3.0 µm ± 0.1 and the median size (d(0.5)) was 2.5 µm ± 0.1, values significantly higher than for the TAG emulsion but still within the observed range for native OBs (Figure 5). We can conclude here that the modification of the process by the addition of an enzymatic hydrolysis step may have slightly decreased the stability of the oil bodies. Indeed, the droplets were still surrounded by proteins, limiting coalescence, but this process clearly modified the internal organization of the emulsion. This re-organization may reflect differences in the ratio of liposoluble and hydrosoluble surfactants, with enzymatic hydrolysis producing additional non-ionic surfactants while the ratio lipid/water is increased. We can assume that the interaction of the droplets with extraneous proteins was sufficiently strong to confer at least a partial resistance to changes in the internal structure of the emulsion but the organization and composition of the interface of the fatty acid emulsion were, thus, clearly different from those of the triglyceride emulsion. Hence, we investigated the differences in the content and composition of non-polar and polar lipids and in the amino-acid distribution of proteins between fatty acid and triglyceride emulsions.

### 2.5. Lipids and Proteins of the Emulsions Obtained

Lipids of the triglyceride emulsion were extracted with the Folch method; however, it appeared that lipids of the fatty acid emulsion could also be extracted with the simple use of ethanol assisted by ultrasound (see materials and methods). This indicates a higher permeability of the droplet membrane, which may be explained by differences in lipid and protein content and composition. The fatty acid profiles of the neutral lipids present in the two emulsions were very similar and differed little from the fatty acid profile of sunflower kernels (Table 3). The integrated process did not, therefore, induce changes in the fatty acid chains.

It is not unusual to obtain different fatty acid profiles between neutral and polar lipids, particularly as phospholipids associate more easily with saturated than with unsaturated chains. This may explain the lower concentration of oleic acid in phospholipids. Several differences were also observed between the phospholipids of the kernel and those of the emulsions (Table 3 and Table 4), which contained larger amounts of saturated chains. It should be borne in mind that the kernel phospholipids include not only those surrounding the oil bodies but also those forming the membranes of the various cells. Furthermore, the phospholipids in cell membranes are generally arranged in a double-layer conformation, whereas those in oil bodies are organized into monolayers, potentially accounting for the differences between kernel phospholipids and emulsion phospholipids. We found no significant difference between the fatty acid profiles of the phospholipids in the FA and TAG emulsions; however, more pronounced differences in phospholipid contents and classes were found between sunflower kernel and emulsions.

The crushing of sunflower seeds in water may lead to the release of a phospholipase. Hence, the phospholipids in the triglyceride emulsion had a higher relative concentration of phosphatidic acid (PA), a product of the hydrolysis of other phospholipids, including phosphatidylcholine (PC) in particular. The phospholipid content of the fatty acid emulsion was much lower, with phosphatidylinositol (PI) undetectable and a higher percentage of phosphatidylethanolamine (PE) than of phosphatidylcholine (PC) (Table 4). During production of the FA emulsion, the medium is incubated for several hours at 43 °C, which may increase the action of the phospholipase enzyme. The lower phospholipid content of the FA emulsion may also be accounted for by the presence of free fatty acids, which may partly replace the phospholipids from the interface.

The percentage of protein in the emulsion was similar for the TAG emulsion (13.1 ± 1.4% *w*/*w* dm) and the FA emulsion (12.0 ± 1.4% *w*/*w* dm)).

Neither emulsion was washed, and therefore they contained both membranous proteins and other proteins surrounding the oil bodies and forming weak or strong interactions with them.

**Figure 6:**
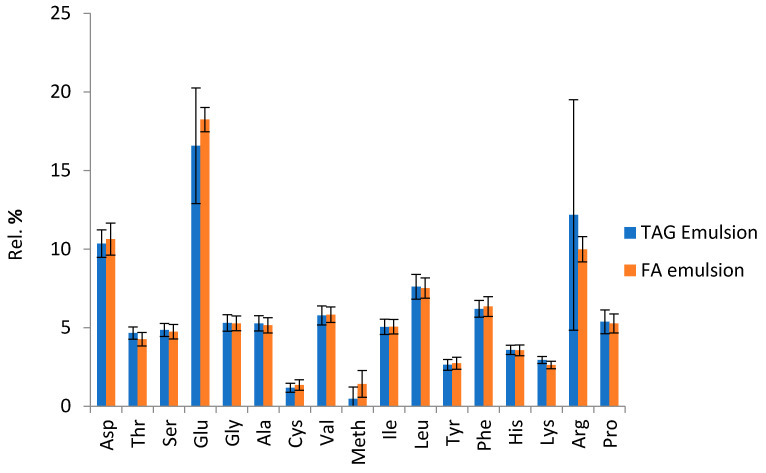
Relative amino-acid composition of the proteins present in the two kinds of emulsions.

The protein compositions of the TAG and FA emulsions were quite similar (Figure 6). The amino-acid composition of the proteins present in the emulsion differed markedly from the usual composition of sunflower oleosins, in which glycine and threonine are more strongly represented [49,51] The difference between the results clearly indicates that the emulsion obtained is not composed of pure oil bodies, but of lipid droplets surrounded by different kinds of proteins. The enzymatic process seems to have little effect on protein composition.

The formation of free fatty acids and partial glycerides modifies the nature of the surfactants of the emulsion. With a lipid/water ratio close to 1 and the simultaneous presence of these new liposoluble surfactants, the emulsion has a complex nature, predominantly O/W and W/O/W (water-in-oil-in-water). The almost disappearance of phospholipids may explain the higher solvent permeability of the droplets.

## 3. Materials and Methods

### 3.1. Materials

Very high oleic sunflower seeds (variety THO) were purchased from Arterris (Lavaur, France). This variety is cultivated in the south of France. The seeds were partially dehulled by an industrial ripple-mill process (Terres Innovia, Pessac, France), and 66% of the initial hulls were discarded. The dehulled kernels had a lipid content of 53.4% with a high oleic acid content of 90.4%, as determined by Gas Chromatography (GC) on the resulting oil. Cyclohexane, chloroform and methanol were purchased from Sigma Aldrich (St Quentin Fallavier, France). *Candida rugosa* enzyme powder (Lipolyve CC at 30,000 AU/g) was purchased from Lyven (Colombelles, France).

### 3.2. Oil-Body Extraction in an Oil-in-Water Triglyceride Emulsion

We added 400 g of partially dehulled sunflower seeds to 1600 g of demineralized water under the action of a high-shear rate device (Silverson L4RT, Silverson Machines Ltd., Waterside, UK) operating at maximum speed (≈5200 rpm) for two 5-min periods separated by a break of 2 min. The mixture was immediately homogenized with a high-pressure homogenizer (Lab 1000, APV, Evreux, France) at 50, 250, and finally 350 bars. The resulting homogenate was stored at 4 °C overnight. It was then split equally between six 500 mL centrifuge bottles and was separated into 3 fractions by centrifugation (Sigma 6K15 Fisher Bioblock Scientific, Illkirch, France) at 10,000× *g* for 10 min at 4 °C. Centrifugation was performed one bottle at a time, and each fraction was collected immediately, to prevent the dissolution of the emulsion in the aqueous phase. For each centrifuge bottle, the aqueous phase was passed through a 100 µm-mesh nylon filter and the emulsion was carefully recovered. The separation resulted in an oil-in-water triglyceride emulsion (top creamy layer), an aqueous phase and a bottom residue. Each fraction was weighed and stored at 4 °C until use.

### 3.3. Oil-Body Extraction in a Complex Fatty Acid Emulsion

We added 400 g of partially dehulled sunflower seeds to 1600 g of demineralized water under the action of a high-shear rate device (Silverson L4RT, Silverson Machines Ltd., Waterside, England) operating at maximum speed (≈5200 rpm) over two five-minute periods separated by a two-minute break. A *Candida rugosa* lipase solution (prepared in advance by dissolving 8 g of enzyme powder in 50 g of demineralized water) was then added. The mixture was immediately homogenized with a high-pressure homogenizer (Lab 1000, APV, Evreux, France) at 50, 250, and finally 350 bars. This step was followed by hydrolysis for 4 h at 43 °C in a constant-temperature reactor, with stirring (1000 rpm). The resulting homogenate was kept at 4 °C overnight and separated into three fractions by centrifugation, as described above.

### 3.4. Determination of the Composition of Each Fraction

As six successive centrifugations were performed, we determined the composition of each fraction six times, to demonstrate the reproducibility of the separation. Dry matter content was determined according to the French NF V03-909 standard. The amount of water and volatile compounds was determined by difference: 100 − (dry matter (%) × 100). Lipid content was determined by accelerated solvent extraction (4 cycles, static phase 10 min, 100 °C, 100 bars) with cyclohexane in a ASE 350 Dionex Thermo apparatus (Dionex, Sunnyvale, CA, US) with the emulsion and bottom residue dry matter as the starting material. The residual lipid content of the aqueous phase was determined as described by E. G. Bligh and W. J. Dyer [52]. Total nitrogen content was determined by elemental analysis on delipidated dry matter for the emulsion and the bottom residue fractions, and on dry matter for the aqueous phase. Protein content was calculated with the standard sunflower nitrogen-to-protein conversion factor of 5.3 ([53]). For clarity, the data are presented as the mean of six determinations for each type of fraction, together with the standard deviation. The complete dataset is available in the Supplementary Materials. The amino-acid composition of the proteins was determined with a Biochrom 20+ amino-acid analyzer (Biochrom Ltd., Cambridge, UK) equipped with a 200 × 4.6 mm column + precolumn system with sodium-based ion-exchange resins.

### 3.5. Extraction of Lipids by Folch’s Method

The dried sample is first crushed (coffee grinder or knife mill) and then mixed with methanol in a 1:10 (*w*/*v*) ratio. The mixture is homogenized for one minute with an Ultraturrax mill. Chloroform is added to reach a ratio of 1:30 (*w*/*v*) solid to solvent and the whole is homogenized for another 2 min. The whole is centrifuged and the supernatant is filtered while the residue is taken up in the same volume of a solution of chloroform/methanol (2/1 *v*/*v*) and homogenized 3 min. The residue is thus treated 3 times. The supernatant phases are collected and represent a volume V. A solution of KCl at 8.8 g/L in water (m/V) of a volume of V/4 is added. The mixture is shaken vigorously and then left to decant. The upper phase is eliminated. The lower phase of a volume V’ is added with a volume V’/4 of a methanol/KCl 8.8 g/L solution (1/1 *v*/*v*). After stirring and decanting, the lower phase is filtered and dried through anhydrous sodium sulfate, then the solvent is evaporated under a nitrogen flow.

### 3.6. Ultrasonic Assisted Extraction of Total Lipids from Fatty Acid Emulsion in Ethanol

Lipids of the fatty acid emulsion can also be extracted with ethanol with ultrasound assistance. Approximately 10 g of fatty acid emulsion is introduced into a plastic Erlenmeyer flask. 150 mL absolute ethanol is added and the ultrasonic probe is immersed in the mixture (VCX750, SONICS Vibra Cell, Sonics & Materials, Newtown, CT, USA equipped with a Ø13 mm fixed tip probe). At room temperature, ultrasound is applied for a total duration of 5 min (5 s ON/10s OFF) with an amplitude of 60%. The solution is then centrifuged at 5000× *g* for 15 min at 20 °C. We obtain a protein pellet and a clear pale yellow organic phase. Ethanol and water are evaporated with a rotary evaporator. The extraction is performed in triplicate. The quantity of lipids extracted is similar to the quantity extracted with Folch extraction while this method is unefficient in the case of the triglyceride emulsion.

### 3.7. Solid Phase Extraction (SPE)

To separate the previously extracted lipids between polar and non-polar molecules, a solid phase extraction method was used. A column was prepared with silica gel ([54]) or a Supelclean LC-Si SPE 500 mg/6 mL cartridge (Supelco, Bellefonte, PA, USA) was used. It is then conditioned by percolating 5 mL of methanol and 5 mL of chloroform through it. 100 mg of lipid extract in 200 µL of chloroform are deposited on the SPE cartridge. After the sample is deposited, it will elute with 5 mL of chloroform to elute the non-polar lipids. Once the fraction is recovered, the elution continues with 10 mL of acetone to elute the glycolipids, and once the second fraction is obtained, with 10 mL of methanol. It is the fraction obtained by this last elution that contains the phospholipids. The methanol is then evaporated under nitrogen flow and the phospholipids are analyzed by HPLC/ELSD chromatography.

### 3.8. Chromatography HPLC/ELSD

After evaporation of methanol, the extract obtained by SPE is taken up in 1 mL of chloroform then possibly diluted according to the phospholipid content and injected (injection volume of 20 µL) in a liquid chromatography apparatus connected to an ELSD (Evaporative Light Scattering Detector) according to the procedure detailed in the literature [55]. The column used is of type Lichrospher 100 diol (5µ) 150 × 3 mm. The oven is set at a temperature of 40 °C and the detector Alltech 3300 (Büchi, Postfach, Switzerland) is operated at a temperature of 35 °C with an air supply at 4 bars and a flow rate of 1.6 L/h. Two eluents are prepared and mixed with a concentration gradient over time (see Supplementary Materials). The different phospholipids are identified by comparing their retention times with those of commercial standards. Quantification is performed by external calibration.

### 3.9. Chromatography GC/FID

The fatty acid profile of phospholipids is determined by gas chromatography coupled with a flame ionization detector. Phospholipids are first hydrolyzed and derivatized by trans-methylation with 0.2 M trimethylsulfonium hydroxide solution in methanol, according to AFNOR EN ISO 152966-3. The fatty acid methyl esters are then analyzed with a gas chromatography equipped with a CP-Select CB column (50 m length, 0.32 mm internal diameter and 0.50 µm film thickness). Helium is used as the carrier gas, at a flow rate of 1.2 mL/min; the injector and the FID detector are maintained at 250 °C. The initial oven temperature was set at 185 °C for 40 min and then raised to 250 °C at a rate of 15 °C/min to be maintained at this value for 10 min.

### 3.10. Measurement of Oil Body Size Distribution

The size distribution of the OBs in emulsions was determined with a Malvern Mastersizer 2000 laser light scattering instrument coupled with a Hydro2000S sample handling unit (Malvern, UK). A fraction of the sample was diluted in the analysis medium and stirred at 3000 rpm. Continuous ultrasound treatment at a tip displacement of 80% was performed until the droplet aggregates were disrupted. Various measurements were performed during the ultrasound treatment. The mean value obtained value is recorded as the final stabilized value.

### 3.11. Cryo-Scanning Electron Microscopy Imaging

Freshly extracted triglyceride and fatty acid emulsions were used to prepare samples for SEM observation. One drop of the emulsion was frozen in nitrogen slush at −220 °C. The frozen sample was transferred, under vacuum, to the cryo-fracture apparatus (Quorum PP3000T Cryo Transfer System, Quorum Technologies, Laughtown, United Kingdom) chamber, in which it was fractured at −145 °C. The temperature was then increased to −95 °C, at which it was maintained for 15 min for sublimation. The sample was then metalized with Pd for 60 s and introduced into the microscope chamber, in which it was maintained at−145 °C during observation, with the electron microscope operating at an accelerating voltage of 5 kV.

### 3.12. Statistical Analysis

Data are presented as the average values of three or more analyses with the standard deviation. Student tests were performed to compare average values. A significance level of α = 0.05 was then used.

## 4. Conclusions

We show here that an integrated aqueous process involving only mechanical steps successfully releases the reserve lipids of sunflower seeds in the form of emulsified native oil bodies. The process we describe here requires only water and mechanical treatments, with no need for exogenous agents such as organic solvents, enzymes or surfactants. The native size of the oil bodies is conserved, as confirmed by laser particle size distribution analysis and scanning electron microscopy. This process is tunable, with transformation steps, such as the use of a recyclable lipase, for example, to mediate the release of the reserve lipids in the form of free fatty acids in a complex emulsion. Both the emulsions obtained were rich in lipids and their protein contents and compositions were similar, but with different phospholipid profiles. These emulsions are naturally stabilized by native surfactants (e.g., proteins or phospholipids), avoiding the need for fastidious oil extraction and formulation steps with extraneous chemical surfactants. The emulsion produced by the initial process, in its natural form (cream) or diluted, may have applications in food formulations, considering the natural resistance of extracted oil bodies against coalescence [37]. The emulsion produced by the modified process including a lipase is rich in free oleic acid and glycerol and could be used in its natural form for more technical applications, such as lubrication [56] or could undergo emulsion breakage to get an oleic-rich fatty acid solution to be used in different industrial sectors as pharmacology [57] or polymer synthesis [58].

## Figures and Tables

**Table 1:** Composition of the triglyceride emulsion, the aqueous phase and the bottom residue obtained with the integrated process.

% (*w*/*w*)	Triglyceride Emulsion	Aqueous Phase	Bottom Residue
Dry matter (%)	46.24 ± 1.18	3.07 ± 0.07	36.77 ± 2.04
Water and volatile compounds (%) ^1^	53.76 ± 1.18	96.93 ± 0.07	63.23 ± 2.04
Ash (%)	0.89 ± 0.07	n.d.	1.15 ± 0.08
Lipid (%)	34.19 ± 2.04	0.14 ± 0.05	22.54 ± 1.19
Total nitrogen (%)	1.17 ± 0.12	0.14 ± 0.01	0.75 ± 0.05
Protein (%) ^2^	6.07 ± 0.65	0.76 ± 0.02	3.98 ± 0.28
Extraneous matter (%)	5.09 ± 1.01	2.18 ± 0.05	9.11 ± 0.79

n.d. = not determined. ^1^ The water and volatile compound content was determined by difference: 100 − (dry matter (%) × 100). ^2^ The protein content was calculated by multiplying total nitrogen content by the standard sunflower nitrogen-to-protein conversion factor of 5.3.

**Table 2:** Composition of the FA emulsion, the aqueous phase and the bottom residue obtained with the modified integrated process, including a lipase.

% (*w*/*w*)	Fatty Acid Emulsion	Aqueous Phase	Bottom Residue
Dry matter (%)	56.99 ± 1.34	4.07 ± 0.07	32.50 ± 1.88
Water and volatile compounds (%) ^1^	43.01 ± 1.34	95.93 ± 0.07	67.50 ± 1.88
Ash (%)	0.48 ± 0.06	<0.1	0.72 ± 0.04
Lipid (%)	39.55 ± 0.76	0.17 ± 0.02	18.46 ± 0.99
Total nitrogen (%)	1.29 ± 0.08	0.15 ± 0.01	0.70 ± 0.03
Protein (%) ^2^	6.82 ± 0.44	0.81 ± 0.02	3.71 ± 0.17
Extraneous matter (%)	10.14 ± 1.55	3.11 ± 0.06	9.61 ± 0.79

^1^ The water and volatile compound content was determined by difference: 100 − (dry matter (%) × 100). ^2^ The protein content was calculated by multiplying the total nitrogen content by the standard sunflower nitrogen-to-protein conversion factor of 5.3.

**Table 3:** Fatty acid composition of the neutral and polar lipids of the kernel, TAG emulsion and FA emulsion.

Fatty Acids (%)	Kernel		TAG Emulsion		FA Emulsion	
	NL	PL	NL	PL	NL	PL
C14:0	0.1 ± 0.0	n.d.	n.d.	n.d.	n.d.	n.d.
C16:0	3.1 ± 0.0	8.5 ± 0.6	3.2 ± 0.0	12.6 ± 1.4	3.3 ± 0.0	10.6 ± 1.1
C18:0	1.4 ± 0.0	2 ± 0.3	1.4 ± 0.0	5.3 ± 1.1	1.5 ± 0.0	5.5 ± 0.5
C20:0	0.1 ± 0.0	n.d.	n.d.	n.d.	n.d.	n.d.
C22:0	0.7 ± 0.0	n.d.	n.d.	n.d.	n.d.	n.d.
SATURATED	5.5 ± 0.0	10.5	4.6 ± 0.0	17.9 ± 2.5	4.8 ± 0.0	16.1 ± 1.6
C16:1n7	0.1 ± 0.0	n.d.	n.d.	n.d.	n.d.	n.d.
C18:1n9	90.9 ± 0.0	84.2 ± 0.9	91.5 ± 0.0	77.2 ± 2.3	92.0 ± 0.0	78.9 ± 2.0
C18:1n7c	1 ± 0.0	0.9 ± 0.0	n.d.	2.2 ± 0.1	n.d.	1.5 ± 0.2
C20:1n9	0.4 ± 0.0	n.d.	n.d.	n.d.	n.d.	n.d.
MONOUNSATURATED	92.4 ± 0.0	85.1 ± 0.9	91.5 ± 0.0	79.4 ± 2.4	92 ± 0.0	80.4 ± 2.2
C18:2n6	2.2 ± 0.0	4.3 ± 0.0	2.2 ± 0.0	2.7 ± 0.1	2.0 ± 0.0	3.5 ± 0.2

n.d. = not detected. NL: neutral lipids. PL: phospholipids.

**Table 4:** Phospholipid content and classes in sunflower seeds and the different emulsions.

Seed and Emulsions	Total (mg/100 g Lipids)	PA (rel %)	PE (rel %)	PC (rel %)	PI (rel %)
Sunflower seed	1811.2 ± 122.2	5.2 ± 0.9	11.6 ± 1.2 a	68 ± 3.7	15.2 ± 2.2 b
TAG emulsion	144.4 ± 6	23.4 ± 1.2	12.4 ± 0.5 a	53.4 ± 3.4	10.8 ± 1.9 b
FA emulsion	17.1 ± 4.4	32 ± 2.2	52.8 ± 0.3	15.2 ± 2.4	n.d.

n.d. = not detected. rel %: relative percentage. a,b: values not significantly different (student test with α = 0.05).

## Data Availability

Not applicable.

## Supplementary Materials

The following are available online at https://www.mdpi.com/article/10.3390/biom12020149/s1, Table S1: Fraction distribution of the integrated process; Table S2: Composition of each batch of triglyceride emulsion; Table S3: Composition of each batch of aqueous phase from the integrated process; Table S4: Composition of each batch of bottom residue obtained with the integrated process; Table S5: Fraction distribution of the modified integrated process, including a lipase; Table S6: Composition of each batch of fatty acid emulsion; Table S7: Composition of each batch of aqueous phase obtained with the modified integrated process, including a lipase; Table S8: Composition of each batch of bottom residue obtained with the modified integrated process including a lipase; Table S9: Details of the eluents used for the chromatographic analysis of phospholipids; Table S10: Elution gradient in HPLC/ELSD chromatography.

## References

[1] Tzen J.T., Lie G.C., Huang A.H. (1992). Characterization of the Charged Components and Their Topology on the Surface of Plant Seed Oil Bodies. J. Biol. Chem..

[2] Tzen J., Huang A. (1992). Surface Structure and Properties of Plant Seed Oil Bodies. J. Cell Biol..

[3] Tzen J., Cao Y., Laurent P., Ratnayake C., Huang A. (1993). Lipids, Proteins, and Structure of Seed Oil Bodies from Diverse Species. Plant Physiol..

[4] Liu C., Hao L., Chen F., Zhu T. (2020). The Mechanism of Extraction of Peanut Protein and Oil Bodies by Enzymatic Hydrolysis of the Cell Wall. J. Oleo Sci..

[5] Deleu M., Vaca-Medina G., Fabre J.-F., Roïz J., Valentin R., Mouloungui Z. (2010). Interfacial Properties of Oleosins and Phospholipids from Rapeseed for the Stability of Oil Bodies in Aqueous Medium. Colloids Surf. B Biointerfaces.

[6] Fetzer A., Herfellner T., Stäbler A., Menner M., Eisner P. (2018). Influence of Process Conditions during Aqueous Protein Extraction upon Yield from Pre-Pressed and Cold-Pressed Rapeseed Press Cake. Ind. Crops Prod..

[7] Latif S., Diosady L.L., Anwar F. (2008). Enzyme-assisted Aqueous Extraction of Oil and Protein from Canola (*Brassica napus* L.) Seeds. Eur. J. Lipid Sci. Technol..

[8] Domínguez H., Sineiro J., Núñez M.J., Lema J.M. (1995). Enzymatic Treatment of Sunflower Kernels before Oil Extraction. Food Res. Int..

[9] Moradi N., Rahimi M. (2019). Effect of Ultrasound- and Pulsed Electric Field-Assisted Enzymatic Treatment on the Recovery and Quality of Sunflower Oil. Sep. Sci. Technol..

[10] Munder S., Latif S., Müller J. (2020). Enzyme-Assisted Aqueous Oil Extraction from High Oleic Sunflower Seeds in a Scalable Prototype Reactor. Waste Biomass Valor.

[11] Aquino D.S., Fanhani A., Stevanato N., Silva C. (2019). Sunflower Oil from Enzymatic Aqueous Extraction Process: Maximization of Free Oil Yield and Oil Characterization. J. Food Process Eng..

[12] Hou L.X., Shang X.L., Wang X., Liu J. (2013). Application of Enzyme in Aqueous Extraction of Sesame Oil. Eur. Food Res. Technol..

[13] Nikiforidis C.V., Kiosseoglou V. (2009). Aqueous Extraction of Oil Bodies from Maize Germ (Zea Mays) and Characterization of the Resulting Natural Oil-in-Water Emulsion. J. Agric. Food Chem..

[14] Souza T.S.P., Dias F.F.G., Koblitz M.G.B., de M. Bell J.M.L.N. (2019). Aqueous and Enzymatic Extraction of Oil and Protein from Almond Cake: A Comparative Study. Processes.

[15] Gibbins R.D., Aksoy H.A., Ustun G. (2012). Enzyme-assisted Aqueous Extraction of Safflower Oil: Optimisation by Response Surface Methodology. Int. J. Food Sci. Technol..

[16] Rosenthal A., Pyle D.L., Niranjan K., Gilmour S., Trinca L. (2001). Combined Effect of Operational Variables and Enzyme Activity on Aqueous Enzymatic Extraction of Oil and Protein from Soybean. Enzym. Microb. Technol..

[17] de Moura J.M.L.N., Campbell K., Mahfuz A., Jung S., Glatz C.E., Johnson L. (2008). Enzyme-Assisted Aqueous Extraction of Oil and Protein from Soybeans and Cream De-Emulsification. J. Am. Oil Chem. Soc..

[18] Campbell K.A., Glatz C.E. (2009). Mechanisms of Aqueous Extraction of Soybean Oil. J. Agric. Food Chem..

[19] Towa L.T., Kapchie V.N., Hauck C., Murphy P.A. (2010). Enzyme-Assisted Aqueous Extraction of Oil from Isolated Oleosomes of Soybean Flour. J. Am. Oil Chem. Soc..

[20] Cheng M.-H., Rosentrater K.A., Sekhon J., Wang T., Jung S., Johnson L.A. (2019). Economic Feasibility of Soybean Oil Production by Enzyme-Assisted Aqueous Extraction Processing. Food Bioprocess Technol..

[21] Zhang Z., Xie Q., Che L. (2020). Synergistic Effects of Ultrasound and Extraction Solvent on the Bioactive Compound in Kenaf Seed Oil. J. Food Sci. Technol..

[22] Amigh S., Taghian Dinani S. (2020). Combination of Ultrasound-Assisted Aqueous Enzymatic Extraction and Cooking Pretreatment for Date Seed Oil Recovery. Heat Mass Transf..

[23] Datt S., Sidhu G.K. (2019). Optimization of Ultrasound Assisted Aqueous Oil Extraction from (*Zea mays* L.) Germ Using Response Surface Methodology. J. Pharmacogn. Phytochem..

[24] Peng L., Ye Q., Liu X., Liu S., Meng X. (2019). Optimization of Aqueous Enzymatic Method for Camellia Sinensis Oil Extraction and Reuse of Enzymes in the Process. J. Biosci. Bioeng..

[25] Liu Z., Gui M., Xu T., Zhang L., Kong L., Qin L., Zou Z. (2019). Efficient Aqueous Enzymatic-Ultrasonication Extraction of Oil from Sapindus Mukorossi Seed Kernels. Ind. Crops Prod..

[26] Tan C.X., Chong G.H., Hamzah H., Ghazali H.M. (2018). Comparison of Subcritical CO_2_ and Ultrasound-Assisted Aqueous Methods with the Conventional Solvent Method in the Extraction of Avocado Oil. J. Supercrit. Fluids.

[27] Goula A.M., Papatheodorou A., Karasavva S., Kaderides K. (2018). Ultrasound-Assisted Aqueous Enzymatic Extraction of Oil from Pomegranate Seeds. Waste Biomass Valor.

[28] Moradi N., Rahimi M., Moeini A., Parsamoghadam M.A. (2018). Impact of Ultrasound on Oil Yield and Content of Functional Food Ingredients at the Oil Extraction from Sunflower. Sep. Sci. Technol..

[29] Jiao J., Li Z.-G., Gai Q.-Y., Li X.-J., Wei F.-Y., Fu Y.-J., Ma W. (2014). Microwave-Assisted Aqueous Enzymatic Extraction of Oil from Pumpkin Seeds and Evaluation of Its Physicochemical Properties, Fatty Acid Compositions and Antioxidant Activities. Food Chem..

[30] Maroušek J. (2013). Use of Continuous Pressure Shockwaves Apparatus in Rapeseed Oil Processing. Clean Technol. Environ. Policy.

[31] Do L.D., Sabatini D.A. (2010). Aqueous Extended-Surfactant Based Method for Vegetable Oil Extraction: Proof of Concept. J. Am. Oil Chem. Soc..

[32] Zhang S., Zhang W., Liu J., Zhao W., Yang R. (2019). Surfactant-Assisted Aqueous Extraction Processing of Camellia Seed Oil by Cyclic Utilization of Aqueous Phase. Eur. J. Lipid Sci. Technol..

[33] Mat Yusoff M., Gordon M.H., Niranjan K. (2015). Aqueous Enzyme Assisted Oil Extraction from Oilseeds and Emulsion De-Emulsifying Methods: A Review. Trends Food Sci. Technol..

[34] Liu W., Xiao B., Yang G., Bi Y., Chen F. (2020). Rapid Salt-Assisted Microwave Demulsification of Oil-Rich Emulsion Obtained by Aqueous Enzymatic Extraction of Peanut Seeds. Eur. J. Lipid Sci. Technol..

[35] White D.A., Fisk I.D., Mitchell J.R., Wolf B., Hill S.E., Gray D.A. (2008). Sunflower-Seed Oil Body Emulsions: Rheology and Stability Assessment of a Natural Emulsion. Food Hydrocoll..

[36] Karkani O.A., Nenadis N., Nikiforidis C.V., Kiosseoglou V. (2013). Effect of Recovery Methods on the Oxidative and Physical Stability of Oil Body Emulsions. Food Chem..

[37] Nikiforidis C.V., Matsakidou A., Kiosseoglou V. (2014). Composition, Properties and Potential Food Applications of Natural Emulsions and Cream Materials Based on Oil Bodies. RSC Adv..

[38] Nikiforidis C.V., Scholten E. (2015). High Internal Phase Emulsion Gels (HIPE-Gels) Created through Assembly of Natural Oil Bodies. Food Hydrocoll..

[39] Nikiforidis C.V., Donsouzi S., Kiosseoglou V. (2016). The Interplay between Diverse Oil Body Extracts and Exogenous Biopolymers or Surfactants. Food Res. Int..

[40] Furse S., Liddell S., Ortori C.A., Williams H., Neylon D.C., Scott D.J., Barrett D.A., Gray D.A. (2013). The Lipidome and Proteome of Oil Bodies from Helianthus Annuus (Common Sunflower). J. Chem. Biol..

[41] Abdullah, Weiss J., Zhang H. (2020). Recent Advances in the Composition, Extraction and Food Applications of Plant-Derived Oleosomes. Trends Food Sci. Technol..

[42] Ding J., Wen J., Wang J., Tian R., Yu L., Jiang L., Zhang Y., Sui X. (2020). The Physicochemical Properties and Gastrointestinal Fate of Oleosomes from Non-Heated and Heated Soymilk. Food Hydrocoll..

[43] Romero-Guzmán M.J., Köllmann N., Zhang L., Boom R.M., Nikiforidis C.V. (2020). Controlled Oleosome Extraction to Produce a Plant-Based Mayonnaise-like Emulsion Using Solely Rapeseed Seeds. LWT.

[44] Fisk I.D., Linforth R.S.T., Taylor A.J., Gray D.A. (2011). Aroma Encapsulation and Aroma Delivery by Oil Body Suspensions Derived from Sunflower Seeds (*Helianthus annus*). Eur Food Res Technol.

[45] Karefyllakis D., Jan van der Goot A., Nikiforidis C.V. (2019). The Behaviour of Sunflower Oleosomes at the Interfaces. Soft Matter.

[46] Matsakidou A., Tsimidou M.Z., Kiosseoglou V. (2019). Storage Behavior of Caseinate-Based Films Incorporating Maize Germ Oil Bodies. Food Res. Int..

[47] Lásztity R., Morsi A.E.E., Samei M.B.A., Ramadan M.E. (1984). Solubility of Sunflower Proteins and Gel Filtration Chromatography of Their Water-Soluble Fractions. Period. Polytech. Chem. Eng..

[48] Iwanaga D., Gray D.A., Fisk I.D., Decker E.A., Weiss J., McClements D.J. (2007). Extraction and Characterization of Oil Bodies from Soy Beans: A Natural Source of Pre-Emulsified Soybean Oil. J. Agric. Food Chem..

[49] Lacey D.J., Wellner N., Beaudoin F., Napier J.A., Shewry P.R. (1998). Secondary Structure of Oleosins in Oil Bodies Isolated from Seeds of Safflower (*Carthamus tinctorius* L.) and Sunflower (*Helianthus annuus* L.). Biochem. J..

[50] Nikiforidis C.V., Kiosseoglou V., Scholten E. (2013). Oil Bodies: An Insight on Their Microstructure—Maize Germ vs. Sunflower Seed. Food Res. Int..

[51] Alexander L., Sessions R., Clarke A., Tatham A., Shewry P., Napier J. (2002). Characterization and Modelling of the Hydrophobic Domain of a Sunflower Oleosin. Planta.

[52] Bligh E.G., Dyer W.J. (1959). A rapid method of total lipid extraction and purification. Can. J. Biochem. Physiol..

[53] Sosulski F.W., Imafidon G.I. (1990). Amino Acid Composition and Nitrogen-to-Protein Conversion Factors for Animal and Plant Foods. J. Agric. Food Chem..

[54] Mills C.T., Goldhaber M.B. (2010). On Silica-Based Solid Phase Extraction Techniques for Isolating Microbial Membrane Phospholipids: Ensuring Quantitative Recovery of Phosphatidylcholine-Derived Fatty Acids. Soil Biol. Biochem..

[55] Silversand C., Haux C. (1997). Improved High-Performance Liquid Chromatographic Method for the Separation and Quantification of Lipid Classes: Application to Fish Lipids. J. Chromatogr. B Biomed. Sci. Appl..

[56] Tan F., Qiao X., Chen J. (2006). Removal of Chemisorbed Lubricant on the Surface of Silver Flakes by Chemicals. Appl. Surf. Sci..

[57] Carrillo Pérez C., Cavia Camarero M.D.M., Alonso de la Torre S. (2012). Antitumor Effect of Oleic Acid; Mechanisms of Action. A Review. Nutr. Hosp..

[58] Lligadas G., Ronda J.C., Galià M., Cádiz V. (2010). Oleic and Undecylenic Acids as Renewable Feedstocks in the Synthesis of Polyols and Polyurethanes. Polymers.

