# Supplementary material for: Aqueous Integrated Process for the Recovery of Oil Bodies or Fatty Acid Emulsions from Sunflower Seeds
Source: Biomolecules. 2022 Jan 18;12(2):149. doi: 10.3390/biom12020149 (PMC8961559; doi:10.3390/biom12020149)
Supplement: Supplementary file 1 [file biomolecules-12-00149-s001.zip › biomolecules-1524498-supplementary.pdf]

*biomolecules*

## Supporting Information

*Article*

# Aqueous Integrated Process for the Recovery of Oil Bodies or Fatty Acid Emulsions from Sunflower Seeds

**Audrey Cassen, Jean-François Fabre *, Eric Lacroux, Muriel Cerny, Guadalupe Vaca Medina, Zéphirin Mouloungui, Othmane Merah and Romain Valentin**

Laboratoire de Chimie Agro-Industrielle (LCA), Université de Toulouse, INRAE, INPT, 31030 Toulouse, France; audrey.cassen@ensiacet.fr (A.C.); eric.lacroux@ensiacet.fr (E.L.); muriel.cerny@ensiacet.fr (M.C.); Guadalupe.VacaMedina@ensiacet.fr (G.V.M.); zephirin.mouloungui@ensiacet.fr (Z.M.); othmane.merah@ensiacet.fr (O.M.); romain.valentin@ensiacet.fr (R.V.)
* Correspondence: jeanfrancois.fabre@ensiacet.fr; Tel.: +33-534323525

## Table of content

Table S1: Fraction distribution of the integrated process ........ 2

Table S2: Composition of each batch of triglyceride emulsion ........ 2

Table S3: Composition of each batch of aqueous phase from the integrated process ........ 2

Table S4: Composition of each batch of bottom residue obtained with the integrated process ........ 3

Table S5: Fraction distribution of the modified integrated process including a lipase ........ 3

Table S6: Composition of each batch of fatty-acid emulsion ........ 3

Table S7: Composition of each batch of aqueous phase obtained with the modified integrated process including a lipase ........ 4

Table S8: Composition of each batch of bottom residue obtained with the modified integrated process including a lipase ........ 4

Table S9: Details of the eluents used for the chromatographic analysis of phospholipids ........ 4

Table S10 : Elution gradient in HPLC/ELSD chromatography ........ 4

**Table S1.** Fraction distribution of the integrated process.

| | Triglyceride Emulsion | Aqueous Phase | Bottom Residue |
|---|---|---|---|
| *Integrated Process n°1* | 7.61 % | 53.98 % | 38.40 % |
| *Integrated Process n°2* | 7.71 % | 53.48 % | 38.82 % |
| ***Average*** | ***7.66 %*** | ***53.73 %*** | ***38.61 %*** |

**Table S2.** Composition of each batch of triglyceride emulsion.

| | *Batch 1* | *Batch 2* | *Batch 3* | *Batch 4* | *Batch 5* | *Batch 6* | **Average** | **S.D.** |
|---|---|---|---|---|---|---|---|---|
| **Dry Matter (%)** | 45.28 | 47.10 | 45.43 | 46.52 | 45.08 | 48.05 | 46.24 | 1.18 |
| **Water and volatiles (%)** | 54.72 | 52.90 | 54.57 | 53.48 | 54.92 | 51.95 | 53.76 | 1.18 |
| **Ash (%)** | 0.80 | 0.99 | 0.98 | 0.86 | 0.84 | 0.90 | 0.89 | 0.07 |
| **Lipids / D.M. (%)** | 70.81 | 77.07 | 76.87 | 70.35 | 71.99 | 76.35 | 73.91 | 3.18 |
| **Lipids (%)** | 32.06 | 36.30 | 34.93 | 32.73 | 32.45 | 36.69 | 34.19 | 2.04 |
| **Total Nitrogen / Deffated D.M. (%)** | 9.95 | 10.23 | 10.11 | 9.33 | 8.35 | 9.21 | 9.53 | 0.71 |
| **Total Nitrogen / D.M. (%)** | 2.90 | 2.34 | 2.34 | 2.77 | 2.34 | 2.18 | 2.48 | 0.29 |
| **Total Nitrogen (%)** | 1.31 | 1.10 | 1.06 | 1.29 | 1.05 | 1.05 | 1.14 | 0.12 |
| **Proteins (%)** | 9.97 | 5.85 | 5.63 | 6.82 | 5.58 | 5.55 | 6.07 | 0.65 |
| **Extraneous Matter (%)** | 5.45 | 3.96 | 3.90 | 6.11 | 6.20 | 4.92 | 5.09 | 1.01 |

D.M. = Dry Matter

S.D. = Standard Deviation

**Table S3.** Composition of each batch of aqueous phase from the integrated process.

| | *Batch 1* | *Batch 2* | *Batch 3* | *Batch 4* | *Batch 5* | *Batch 6* | **Average** | **S.D.** |
|---|---|---|---|---|---|---|---|---|
| **Dry Matter (%)** | 3.00 | 3.06 | 3.04 | 3.07 | 3.08 | 3.20 | 3.07 | 0.07 |
| **Water and volatiles (%)** | 97.00 | 96.94 | 96.96 | 96.93 | 96.92 | 96.80 | 96.93 | 0.07 |
| **Lipids (%)** | 0.09 | 0.12 | 0.18 | 0.08 | 0.20 | 0.17 | 0.14 | 0.05 |
| **Total Nitrogen / D.M. (%)** | 4.63 | 4.68 | 4.60 | 4.69 | 4.59 | 4.66 | 4.64 | 0.04 |
| **Total Nitrogen (%)** | 0.14 | 0.14 | 0.14 | 0.14 | 0.14 | 0.15 | 0.14 | 0.01 |
| **Proteins (%)** | 0.74 | 0.76 | 0.74 | 0.76 | 0.75 | 0.79 | 0.76 | 0.02 |
| **Extraneous Matter (%)** | 2.17 | 2.18 | 2.12 | 2.22 | 2.13 | 2.24 | 2.18 | 0.05 |

D.M. = Dry Matter

S.D. = Standard Deviation

**Table S4.** Composition of each batch of bottom residue obtained with the integrated process.

| | *Batch 1* | *Batch 2* | *Batch 3* | *Batch 4* | *Batch 5* | *Batch 6* | **Average** | **S.D.** |
|---|---|---|---|---|---|---|---|---|
| **Dry Matter (%)** | 36.43 | 38.61 | 35.31 | 36.54 | 34.12 | 39.61 | 36.77 | 2.04 |
| **Water and volatiles (%)** | 63.57 | 61.39 | 64.69 | 63.46 | 65.88 | 60.39 | 63.23 | 2.04 |
| **Ash (%)** | 1.15 | 1.15 | 1.16 | 1.21 | 1.00 | 1.21 | 1.15 | 0.08 |
| **Lipids / D.M. (%)** | 63.24 | 61.09 | 62.06 | 60.77 | 60.51 | 60.15 | 61.30 | 1.15 |
| **Lipids (%)** | 23.04 | 23.58 | 21.91 | 22.21 | 20.65 | 23.82 | 22.54 | 1.19 |
| **Total Nitrogen / Deffated D.M. (%)** | 5.85 | 5.30 | 5.15 | 5.00 | 5.23 | 5.15 | 5.28 | 0.30 |
| **Total Nitrogen / D.M. (%)** | 2.15 | 2.06 | 1.95 | 1.96 | 2.06 | 2.05 | 2.04 | 0.07 |
| **Total Nitrogen (%)** | 0.78 | 0.80 | 0.69 | 0.72 | 0.70 | 0.81 | 0.75 | 0.05 |
| **Proteins (%)** | 4.15 | 4.22 | 3.65 | 3.80 | 3.73 | 4.31 | 3.98 | 0.28 |
| **Extraneous Matter (%)** | 8.09 | 9.65 | 8.58 | 9.33 | 8.75 | 10.27 | 9.11 | 0.79 |

D.M. = Dry Matter

S.D. = Standard Deviation

**Table S5.** Fraction distribution of the modified integrated process including a lipase.

| | **Triglyceride Emulsion** | **Aqueous Phase** | **Bottom Residue** |
|---|---|---|---|
| *Enzymatically-modified Integrated Process n°1* | 9.29 % | 53.61 % | 37.09 % |
| *Enzymatically-modified Integrated Process n°2* | 9.34 % | 56.32 % | 34.34 % |
| ***Average*** | ***9.32 %*** | ***54.97 %*** | ***35.72 %*** |

**Table S6.** Composition of each batch of fatty-acid emulsion.

| | *Batch 1* | *Batch 2* | *Batch 3* | *Batch 4* | *Batch 5* | *Batch 6* | **Average** | **S.D.** |
|---|---|---|---|---|---|---|---|---|
| **Dry Matter (%)** | 57.86 | 58.10 | 57.42 | 57.97 | 55.33 | 55.24 | 56.99 | 1.34 |
| **Water and volatiles (%)** | 42.14 | 41.90 | 42.58 | 42.03 | 44.67 | 44.76 | 43.01 | 1.34 |
| **Ash (%)** | 0.57 | 0.51 | 0.49 | 0.46 | 0.46 | 0.41 | 0.48 | 0.06 |
| **Lipids / D.M. (%)** | 66.10 | 67.73 | 68.85 | 69.97 | 71.79 | 72.17 | 69.44 | 2.35 |
| **Lipids (%)** | 38.25 | 39.35 | 39.54 | 40.56 | 39.72 | 39.87 | 39.55 | 0.76 |
| **Total Nitrogen / Deffated D.M. (%)** | 7.29 | 6.34 | 7.04 | 7.51 | 7.89 | 8.49 | 7.42 | 0.74 |
| **Total Nitrogen / D.M. (%)** | 2.47 | 2.04 | 2.19 | 2.25 | 2.23 | 2.36 | 2.26 | 0.15 |
| **Total Nitrogen (%)** | 1.43 | 1.19 | 1.26 | 1.31 | 1.23 | 1.31 | 1.29 | 0.08 |
| **Proteins (%)** | 7.57 | 6.29 | 6.67 | 6.93 | 6.53 | 6.92 | 6.82 | 0.44 |
| **Extraneous Matter (%)** | 11.47 | 11.95 | 10.73 | 10.01 | 8.62 | 8.05 | 10.14 | 1.55 |

D.M. = Dry Matter

S.D. = Standard Deviation

**Table S7.** Composition of each batch of aqueous phase obtained with the modified integrated process including a lipase.

| | *Batch 1* | *Batch 2* | *Batch 3* | *Batch 4* | *Batch 5* | *Batch 6* | **Average** | **S.D.** |
|---|---|---|---|---|---|---|---|---|
| **Dry Matter (%)** | 4.05 | 4.07 | 4.06 | 4.20 | 4.07 | 4.00 | 4.07 | 0.07 |
| **Water and volatiles (%)** | 95.95 | 95.93 | 95.94 | 95.80 | 95.93 | 96.00 | 95.93 | 0.07 |
| **Lipids (%)** | 0.16 | a.v. | 0.16 | 0.18 | 0.19 | a.v. | 0.17 | 0.02 |
| **Total Nitrogen / D.M. (%)** | 3.70 | 3.72 | 3.71 | 3.71 | 3.85 | 3.72 | 3.73 | 0.06 |
| **Total Nitrogen (%)** | 0.15 | 0.15 | 0.15 | 0.16 | 0.16 | 0.15 | 0.15 | 0.01 |
| **Proteins (%)** | 0.79 | 0.80 | 0.80 | 0.82 | 0.83 | 0.79 | 0.81 | 0.02 |
| **Extraneous Matter (%)** | 3.10 | n.c. | 3.10 | 3.19 | 3.05 | n.c. | 3.11 | 0.06 |

D.M. = Dry Matter

S.D. = Standard Deviation

a.v. = Abnormal value

n.c. = Not calculated

**Table S8.** Composition of each batch of bottom residue obtained with the modified integrated process including a lipase.

| | *Batch 1* | *Batch 2* | *Batch 3* | *Batch 4* | *Batch 5* | *Batch 6* | **Average** | **S.D.** |
|---|---|---|---|---|---|---|---|---|
| **Dry Matter (%)** | 30.82 | 35.36 | 32.84 | 33.97 | 31.13 | 30.89 | 32.50 | 1.88 |
| **Water and volatiles (%)** | 69.18 | 64.64 | 67.16 | 66.03 | 68.87 | 69.11 | 67.50 | 1.88 |
| **Ash (%)** | 0.79 | 0.74 | 0.71 | 0.72 | 0.70 | 0.66 | 0.72 | 0.04 |
| **Lipids / D.M. (%)** | 56,62 | 56.80 | 57,17 | 55.70 | 57.36 | 57.19 | 56.81 | 0.61 |
| **Lipids (%)** | 17.45 | 20.08 | 18.77 | 18.92 | 17.86 | 17.67 | 18.46 | 0.99 |
| **Total Nitrogen / Deffated D.M. (%)** | 5.00 | 4.88 | 4.98 | 4.75 | 4.94 | 5.42 | 4.99 | 0.23 |
| **Total Nitrogen / D.M. (%)** | 2.17 | 2.11 | 2.13 | 2.10 | 2.11 | 2.32 | 2.15 | 0.08 |
| **Total Nitrogen (%)** | 0.67 | 0.74 | 0.70 | 0.71 | 0.66 | 0.72 | 0.70 | 0.03 |
| **Proteins (%)** | 3.54 | 3.95 | 3.71 | 3.79 | 3.48 | 3.80 | 3.71 | 0.17 |
| **Extraneous Matter (%)** | 9.05 | 10.59 | 9.64 | 10.55 | 9.10 | 8.77 | 9.61 | 0.79 |

D.M. = Dry Matter

S.D. = Standard Deviation

**Table S9.** Details of the eluents used for the chromatographic analysis of phospholipids.

| Eluent A | | Eluent B | |
|---|---|---|---|
| Isooctane | 82% | Isopropanol | 85% |
| Isopropanol | 17% | Eau | 14% |
| Acetic acid | 1% | Acetic acid | 1% |
| Triethylamine | 0,08% | Triethylamine | 0,08% |

**Table S10.** : Elution gradient in HPLC/ELSD chromatography.

| | Time | | | | | |
|---|---|---|---|---|---|---|
| | 0 | 24’ | 25’ | 32’ | 33’ | 40’ |
| % A | 95 | 60 | | | 95 | 95 |
| % B | 5 | 40 | 100 | 100 | 5 | 5 |
